# DOP Stimulates Heterotrophic Bacterial Production in the Oligotrophic Southeastern Mediterranean Coastal Waters

**DOI:** 10.3389/fmicb.2019.01913

**Published:** 2019-08-16

**Authors:** Guy Sisma-Ventura, Eyal Rahav

**Affiliations:** Israel Oceanographic and Limnological Research, National Institute of Oceanography, Haifa, Israel

**Keywords:** organic nutrients, DOP, southeastern Mediterranean Sea, P-turnover time, bacterial production, primary production

## Abstract

Phytoplankton and heterotrophic bacteria rely on a suite of inorganic and organic macronutrients to satisfy their cellular needs. Here, we explored the effect of dissolved inorganic phosphate (PO_4_) and several dissolved organic molecules containing phosphorus [ATP, glucose-6-phosphate, 2-aminoethylphosphonic acid, collectively referred to as dissolved organic phosphorus (DOP)], on the activity and biomass of autotrophic and heterotrophic microbial populations in the coastal water of the southeastern Mediterranean Sea (SEMS) during summertime. To this end, surface waters were supplemented with PO_4_, one of the different organic molecules, or PO_4_ + ATP, and measured the PO_4_ turnover time (Tt), alkaline phosphatase activity (APA), heterotrophic bacterial production (BP), primary production (PP), and the abundance of the different microbial components. Our results show that PO_4_ alone does not stimulate any significant change in most of the autotrophic or heterotrophic bacterial variables tested. ATP addition (alone or with PO_4_) triggers the strongest increase in primary and bacterial productivity or biomass. Heterotrophic bacterial abundance and BP respond faster than phytoplankton (24 h post addition) to the various additions of DOP or PO_4_ + ATP, followed by a recovery of primary productivity (48 h post addition). These observations suggest that both autotrophic and heterotrophic microbial communities compete for labile organic molecules containing P, such as ATP, to satisfy their cellular needs. It also suggests that SEMS coastal water heterotrophic bacteria are likely C and P co-limited.

## Introduction

Large parts of the global oceans are considered low nutrient low chlorophyll (LNLC) areas, generally characterized by a deficiency in macronutrients such as P, N, and C (Tyrrell, 1999). Such LNLC regions exhibit a low chlorophyll-*a* biomass (Uitz et al., 2010), dominance of small-size microbial communities (pico- and nano-phytoplankton and heterotrophic bacteria) (Yacobi et al., 1995), and low primary production (PP) rates (Marañón et al., 2010). The surface waters of the southeastern Mediterranean Sea (SEMS) are considered to be a LNLC ultra-oligotrophic system (Siokou-Frangou et al., 2010; Pujo-Pay et al., 2011; Rahav et al., 2013), where PO_4_ is the main limiting factor for heterotrophic microbial activity (Krom et al., 1991, 2014; Thingstad et al., 2005) while during summer NO_3_ + PO_4_ limit phytoplankton (Kress et al., 2005; Zohary et al., 2005). The coastal waters of the SEMS are also considered oligotrophic with low algal biomass (chlorophyll-*a*) levels (Berman et al., 1984; Azov, 1986; Herut et al., 2000) and low PP rates (Raveh et al., 2015; Rahav et al., 2018), despite being exposed to various anthropogenic stressors (Frank et al., 2017; Rahav and Bar-Zeev, 2017; Grossowicz et al., 2019; Kress et al., 2019; Raveh et al., 2019).

A recent inorganic-nutrient-addition study was conducted in the SEMS’s Israeli coastal waters, which found that cyanobacteria are mainly NO_3_-limited, whereas NO_3_ or Si(OH)_4_ (or both) limit pico-eukaryotes (Rahav et al., 2018). Rahav et al. also demonstrated that heterotrophic bacteria are generally not limited by PO_4_, contrary to the open waters of the SEMS (Krom et al., 2005; Thingstad et al., 2005). Some of the anthropogenic pollutants regularly discharged along the coastal SEMS contain organic substances such as phosphonates (Belkin et al., 2017; Petersen et al., 2018) and treated sewage (Kress et al., 2016; Rahav and Bar-Zeev, 2017) that can fuel microbial activity (Dyhrman et al., 2006).

Surface PO_4_ levels along the SEMS coast are usually below the detection threshold (Kress et al., 2014; Raveh et al., 2015). Therefore, we hypothesized that surface-water microbes inhabiting the SEMS will likely use dissolved organic phosphorus (DOP) as a P source for various metabolic activities in addition to PO_4_ (Van Wambeke et al., 2002; Rofner et al., 2016), depending on each specific source’s availability. Due to the rapid biological uptake of PO_4_, the upper ocean phosphorus pool is usually dominated by organic forms. This organic P pool is utilized by several organisms using specific enzymes, like the alkaline phosphatase (APase) that allows bacteria to access the P-containing organic molecules, specifically where PO_4_ is present in limited concentrations (Karl and Björkman, 2015). Approximately 50% of open ocean microbes have the cellular machinery to utilize DOP by APase (Sebastián and Ammerman, 2011). Most organic phosphorus produced by photoautotrophs is later degraded by heterotrophic bacteria, where some molecules, such as phosphonates, are degraded by the enzyme C–P lyase. The production and degradation of organic phosphorus is therefore linked to that of organic carbon (Martínez-García et al., 2010; Sebastián and Ammerman, 2011).

Studies show that DOP is an important P source for marine primary producers and bacteria in many LNLC ecosystems (Dyhrman et al., 2006; Karl and Björkman, 2014; Björkman et al., 2018), including the Eastern Mediterranean Sea (Djaoudi et al., 2018). DOP comprise ∼40% of the P pool in the Eastern Mediterranean Sea (ranging between 15 and 60 nmol L^–1^) (Pujo-Pay et al., 2011; Djaoudi et al., 2018). Furthermore, recent mass balance calculations suggested that the Mediterranean Sea is a net heterotrophic system, where DOP supports a significant portion of the new primary production (Powley et al., 2017). Little is known yet about the role of different organic species of P (as well as N and C) in regulating the production of heterotrophic or autotrophic bacteria of the SEMS in general, and particularly in its coastal waters (Sebastián et al., 2012).

The objective of this study was to examine whether phytoplankton and heterotrophic bacteria are affected by the availability of different DOP species during summertime in the SEMS’s coastal waters. We hypothesized that during summertime, when the most oligotrophic conditions prevail (and thus PO_4_ levels are close-to or below detection), DOP may be an important P source for microbial communities.

## Materials and Methods

Surface (∼0.5 m) SEMS coastal water was collected (Tel Shikmona, Haifa, 32°49′34N, 34°57′20 E) in acid-washed transparent Nalgene bottles during the summer (June 10–13, 2018), pre-filtered to remove large-size zooplankton (125 μm, Kress et al., 2005; Rahav et al., 2018), and placed in a land-based tank filled with coastal water to maintain ambient temperature (28.7–29.6°C, WTW multiline 3410) and light (210–19,930 Lux, Onset UA-002-64) conditions (Rahav et al., 2016). All microcosm bottles were run in triplicates. Microcosm bioassays (10 L each) included: 500 nmol L^–1^ K_2_HPO_4_ (PO_4_, Merck 119898), 100–500 nmol L^–1^ of various DOP molecules that are considered the main sources of P to primary and bacterial production in the study area (Sebastián et al., 2012), such as: Adenosine 3-Phosphate (ATP, 100 nmol L^–1^, Sigma-Aldrich A9272); glucose 6-phosphate (G6P, 500 nmol L^–1^, Sigma-Aldrich G7879); 2-aminoethylphosphonic acid (2-AEPn, 500 nmol L^–1^, Sigma-Aldrich 268674) and controls. Additional triplicate bottles were also supplemented with PO_4_ and ATP (i.e., combined addition, same concentrations as above, 500 nmol L^–1^ PO_4_ and 100 nmol L^–1^ ATP). In order to represent a realistic scenario of anthropogenically affected coastal waters, we tested DOP concentrations similar to those measured along the SEMS (measured as total P, Ashkelon; Kress et al., 2019). Sub-samples were collected from the microcosm bottles at 0, 4, 24, and 48 h post addition, and were tested for microbial responses as described below.

Additions were chosen in an attempt to relief P limitation on one hand [half of the addition tested in Martínez-García et al. (2010) and Tsiola et al. (2017), twice the maximal value reported for the same area during winter, Kress et al. (2005)], and to address the heterogeneous nature of the DOP pool that consists of many P species on the other hand (Karl and Björkman, 2014; Rofner et al., 2016; Björkman et al., 2018). The selected P molecules represent the main groups of DOP in seawater: P-nucleotides, monophosphate esters, and phosphonates. Each is synthesized by different enzymes (Karl and Björkman, 2015). They also represent the different ways P bonds in both C-P (6:1, G6P) and C-P-N (3.3:1.7:1, ATP; 1:1:2 for 2-AEPn). The DOP additions were also selected to address the different saturation state of P in ATP and G6P, as described in Rofner et al. (2016), where G6P uptake increased with further additions without reaching saturation, while ATP uptake fluctuated without a clear pattern. This suggests that high concentrations of G6P (and likely of 2-AEPn as well) could be utilized by heterotrophic bacterial communities without reaching saturation.

### Inorganic and Organic Nutrients

Nutrient concentrations were determined in a three-channel segmented flow auto-analyzer system (AA-3 Seal Analytical) following Kress et al. (2014). The limit of detection (LOD), estimated as three times the standard deviation of 10 measurements of the blank (low nutrient seawater collected from the off-shore SEMS), was 8 nmol L^–1^ for PO_4_, 50 nmol L^–1^ for total dissolved phosphorus (TDP) and Si(OH)_4_, and 80 nmol L^–1^ for NO_2_ + NO_3_ (NO_*x*_). The reproducibility of the analyses was determined using certified references martials (CRM): MOOS 3 (PO_4_, NO_*x*_, and Si(OH)_4_), VKI 4.1 (NO_*x*_), and VKI 4.2 [PO_4_ and Si(OH)_4_]. Results were accepted when measured CRM’s were within ± 5% from the certified values. TDP concentrations were measured on filtered (Minisart^®^ 0.45 μm) samples following potassium persulfate digestion and ultraviolet (UV) photo-oxidation, using a digestion block system (Seal Analytical). Reproducibility of the analyses were examined with VKI 4.2 and Deep Sea Reference (DSR) material. DOP concentrations were determined by subtracting PO_4_ from TDP concentrations. The consumption rate of each nutrient was estimated according to the following ratio (*T*_0_ – *T*_final._)/time.

### Pico- and Nano-Phytoplankton and Heterotrophic Bacterial Abundance

Samples (1.8 ml) were fixed with glutaraldehyde (0.02% v:v, Sigma-Aldrich G7651), frozen in liquid nitrogen, and stored at −80°C until analysis within 2 weeks. *Synechococcus* and *Prochlorococcus* (hereafter collectively referred to as cyanobacteria), autotrophic pico- and nano-eukaryotes (maximal size ∼70 μm), and heterotrophic bacterial abundances were determined using an Attune^®^ Acoustic Focusing Flow Cytometer (Applied Biosystems) as described in Bar-Zeev and Rahav (2015).

### Primary Production (PP)

Samples (50 ml) were spiked with 5 μCi of radiolabeled NaH^14^CO_3_ (Perkin Elmer, specific activity 56 mCi mmol^–1^) and incubated in the same pool as the microcosm bottles for 4–5 h under natural sunlight and *in situ* temperature conditions (Steemann-Nielsen, 1952). The incubations were terminated by filtering the seawater through glass fiber filters (GF/F, Millipore, 0.7-μm pore size) at low pressure (∼50 mmHg). Measurements of the added activity and of dark controls were also performed. The filters were placed overnight in scintillation plastic vials containing 50 μL of 32% HCl, and then 5 ml of a scintillation cocktail (Ultima Gold) were added. Radioactivity was measured using a TRI-CARB 2100 TR (Packard) liquid scintillation counter.

### Bacterial Production (BP)

Bacterial production was estimated using the ^3^H-leucine incorporation method (Perkin Elmer, specific activity 123 Ci mmol^–1^) followed by micro-centrifugation (Simon et al., 1990). Samples (1.7 ml) were incubated with 10 nmol leucine L^–1^ for 4–5 h under ambient temperature in the dark. Triplicate additions of trichloroacetic acid (TCA) were performed at each time-point and served as controls. The incubations were terminated with 100 μL of concentrated (100%) cold TCA. After adding 1 mL of scintillation cocktail (Ultima-Gold) to each vial, the samples were counted using a TRI-CARB 2100 TR (Packard) liquid scintillation counter. A conversion factor of 3 kg C mol^–1^ Leu^–1^ incorporated was used, assuming an isotopic dilution of 2.0 (Simon and Azam, 1989). We chose using this theoretical conversion factor as has been reported in numerous studies from the SEMS for comparison (e.g., Tanaka et al., 2007; Raveh et al., 2015; Rahav and Bar-Zeev, 2017). It is to be noted, though, that a lower conversion factor (e.g., 0.5–1.0 kg C mol Leu^–1^) was applied in other oligotrophic regimes (Teira et al., 2015). This issue should be investigated in the oligotrophic SEMS waters using empirical approaches in future studies.

### PO_4_ Turnover Time

Phosphate turnover time (Tt) was measured using carrier-free ^33^PO_4_ (Perkin Elmer, specific activity: 40–158 Ci mg^–1^) as described in Tanaka et al. (2011). Radioisotopes (final concentration 20–40 pmol L^–1^) were added to 10 ml of triplicate samples and incubated in the dark for 10 min. Subsamples (0.1 mL) were collected to measure the added radioactivity. Following a 20 min incubation samples were filtered onto 0.2 μm polycarbonate filters supported by glass fiber filters saturated with 1 mmol L^–1^ of KH_2_PO_4_. Background levels were measured using samples fixed with ultra-pure 50 μl glutaraldehyde (Sigma-Aldrich G7651) before isotope addition, and values were subtracted from the sample reads. After adding 2 mL of scintillation cocktail (Ultima Gold) the samples were radio-assayed and counted using a TRI-CARB 2100 TR (Packard) liquid scintillation counter.

### Alkaline Phosphatase Activity

Seawater subsamples (1 ml) were collected from each microcosm bottle in triplicates, to which fluorescent substrate MUF-P was added (methylumbelliferone-phosphate, final concentration 0.1 μM, Sigma M8168), and incubated in the dark (covered with aluminum foil) for 4–8 h under ambient temperature. The reaction was terminated with 100 μl of 1 M ultra-pure NaOH (Sigma 79724). Alkaline phosphatase activity (APA) was detected following the hydrolysis of MUF-P to MUF, resulting in a highly fluorescent product that was measured with a spectro-fluorometer (excitation-364 nm and emission-448 nm). A standard curve with MUF (Sigma M1508) was used to quantify the amount of MUF produced by APA (Belkin et al., 2017).

### Statistical Analyses

The data in the figures and table are averages and standard deviations (*n* = 3). The differences between treatments were analyzed using analysis of variance (ANOVA) and FISHER *post hoc* test, with *p*-value of 0.05. Data was first log-transformed to ensure homogeneity. All tests were performed using the XLSTAT software.

## Results

### Initial Water Characteristics

The seawater collected from the SEMS coast exhibited low nutrient concentrations, typical to oligotrophic basins (Table 1). Inorganic nutrient levels were generally low, especially PO_4_ (49 ± 26 nmol L^–1^), while DOP reached 4-fold higher concentrations (118 ± 22 nmol L^–1^). NO_2_ + NO_3_ concentration averaged 453 ± 237 nmol L^–1^, resulting in an N:P ratio of ∼9:1. APA rates were relatively low (7.6 ± 0.7 nmol MUF L^–1^ h^–1^), and Tt was relatively high (11.8 ± 2.2 h), compared to previously measured levels in the P-limited offshore waters of the Eastern Mediterranean Sea (Tanaka et al., 2007, 2011; Sebastián et al., 2012). In terms of abundance, small-size cyanobacteria and heterotrophic bacteria dominated the microbial community (∼8.5 × 10^4^ and ∼8.3 × 10^5^ cells ml^–1^, respectively), whereas autotrophic algae (i.e., pico- and nano-phytoplankton) were scarce (2.0 × 10^3^ cells ml^–1^).

**TABLE 1:** Initial characteristics of the seawater used in the microcosm experiment.

**Variable**	**unit**	**Average ± SD**
NO_2_ + NO_3_	nmol L^–1^	453 ± 237
PO_4_	nmol L^–1^	49 ± 26
Si(OH)_4_	nmol L^–1^	1566 ± 19
DOP	nmol L^–1^	118 ± 22
Cyanobacteria	cells × 10^4^ ml^–1^	8.5 ± 4.0
Pico- and nano-eukaryotes	cells × 10^3^ ml^–1^	2.0 ± 0.0
Heterotrophic bacteria	cells × 10^5^ ml^–1^	8.3 ± 9.7
APA	nmol MUF L^–1^ h^–1^	7.6 ± 0.7
P turnover-time	h	11.8 ± 2.2
Bacterial production	μg C L^–1^ h^–1^	1.5 ± 0.1
Primary production	μg C L^–1^ h^–1^	2.4 ± 1.1

*Values presented are the averages and corresponding standard deviation from triplicate samples.*

### Response to DIP or DOP Additions

**FIGURE 1:**
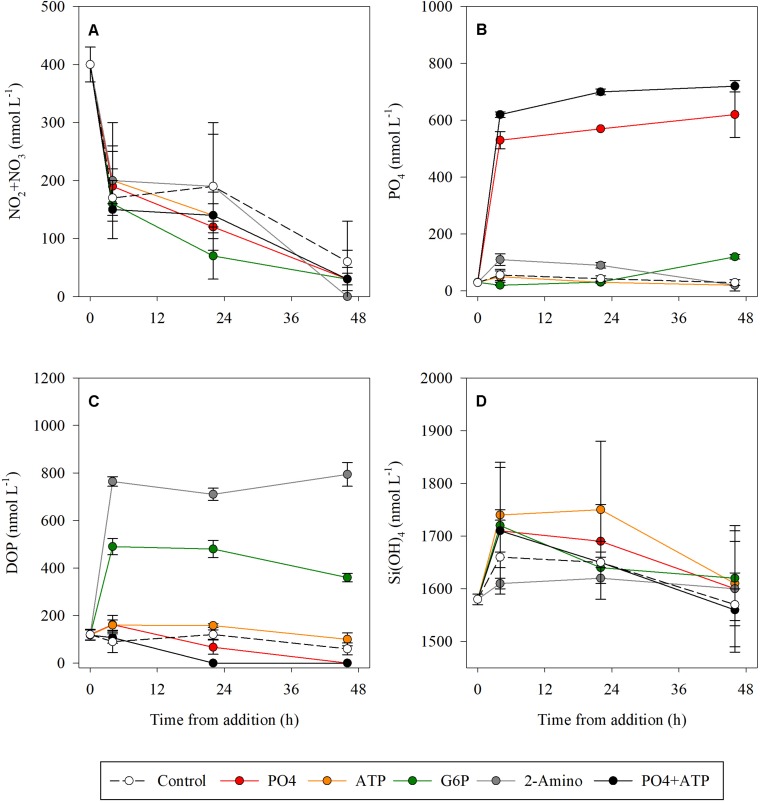
Temporal dynamics of NO_2_ + NO_3_ **(A)**, PO_4_ **(B)**, DOP **(C)**, and Si(OH)_4_ **(D)** following the addition of PO_4_ (red), ATP (orange), G6P (green), 2-AEPn (gray), PO_4_ + ATP (black), and un-amended controls (white). Values presented are the average and corresponding standard deviation (*n* = 3).

All treatments ended with NO_3_ + NO_2_ levels below the detection values, compared to only 30% of the initial concentration measured in the controls (Figure 1A and Supplementary Table S1). PO_4_ levels remained constant throughout the duration of the experiment in both the PO_4_-amended microcosms and the PO_4_ + ATP addition treatments (Figure 1B and Supplementary Table S1), while a reduction was measured in the ATP (57%) and the 2-2-AEPn (81%) bioassays (Figure 1). DOP levels were below detection level (100% reduction) in the PO_4_ and PO_4_ + ATP treatments 48 h post addition (Figure 1C and Supplementary Table S1). Reduction in DOP levels was observed post addition of ATP (37%) and G6P (26%), while remaining unchanged post addition of 2-AEPn. Si(OH)_4_ concentration was reduced by <10% in all treatments and controls (Figure 1D and Supplementary Table S1).

**FIGURE 2:**
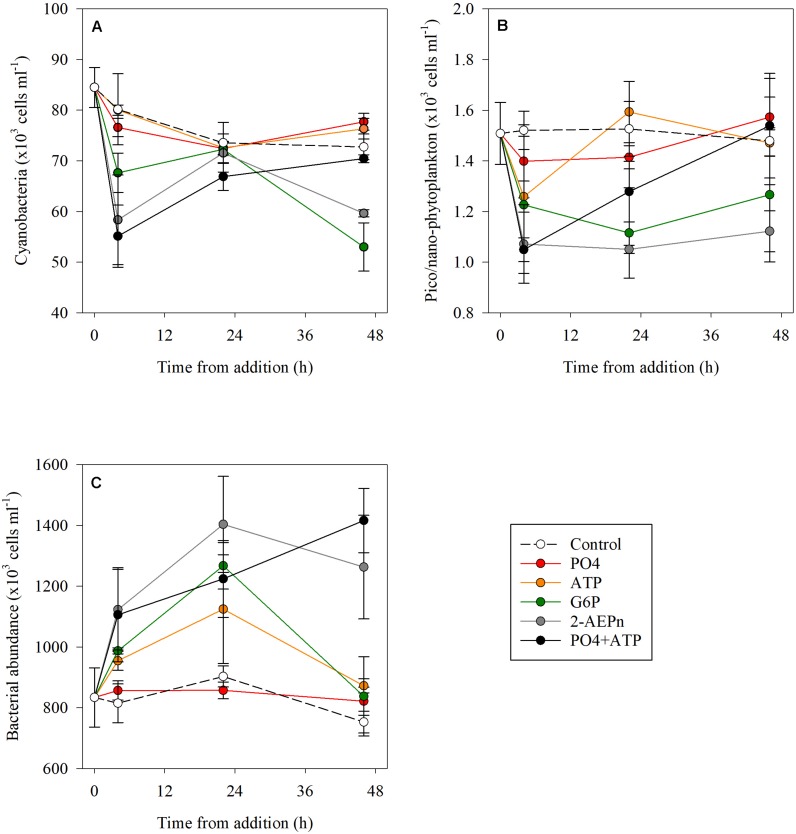
Temporal dynamics of cyanobacterial abundance **(A)**, pico- and nano-eukaryote abundance **(B)**, and heterotrophic bacterial abundance **(C)** following the addition of PO_4_ (red), ATP (orange), G6P (green), 2-AEPn (gray), PO_4_ + ATP (black), and un-amended controls (white). Values presented are the average and corresponding standard deviation (*n* = 3). Note the different *Y* axis.

Cyanobacterial abundance was significantly lower (15–30%) at T_4_ in the PO_4_ + ATP, 2-2-AEPn and G6P treatment, compared to the controls (*p* < 0.01), while no change was detected in the ATP or PO_4_ bottles. Cyanobacterial abundance in the combined treatment (PO_4_ + ATP), the PO_4_ and ATP additions, returned to “typical” (control) values at T_48_ (*p* = 0.68), but were lower by 26 and 18% in the G6P and 2-AEPn treatments, respectively (Figure 2A and Supplementary Table S1). Similarly, pico- and nano-eukaryotic abundance was lower by 8–30% in all treatments compared to the controls (*p* < 0.05) at the beginning of the measurements (T_4_) (Figure 2B). At the conclusion of the experiment (T_48_), pico- and nano-eukaryotes abundance was similar to the controls in the PO_4_ or ATP treatments (alone and combined) (Figure 2B), and were lower by 14–24% in the G6P and 2-AEPn treatments. Contrary to the autotrophic microbes (cyanobacteria and pico- and nano-eukaryotes), heterotrophic bacterial abundance significantly increased in all DOP treatments (maximal 68%, *p* = 0.01) and PO_4_ + ATP (maximal 88%, *p* = 0.01) additions, while bacterial abundance in the PO_4_ addition remained unchanged (*p* = 0.36) (Figure 2C).

**FIGURE 3:**
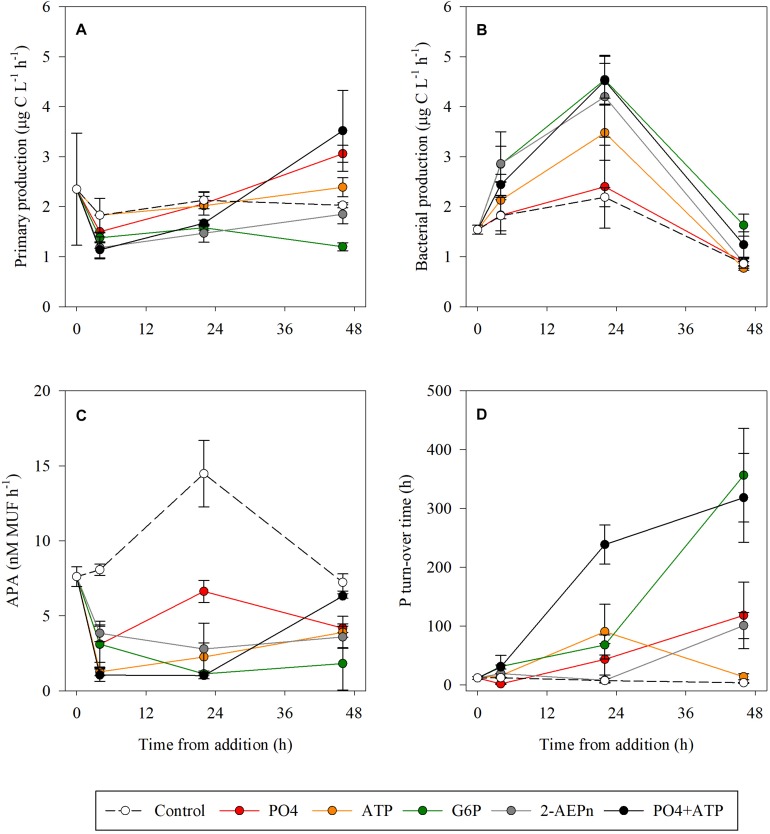
Temporal dynamics of primary production **(A)**, bacterial production **(B)**, alkaline phosphatase activity **(C)**, and P turnover time **(D)** following the addition of PO_4_ (red), ATP (orange), G6P (green), 2-AEPn (gray), PO_4_ + ATP (black), and un-amended controls (white). Values presented are the average and corresponding standard deviation (*n* = 3).

As for autotrophic microbial abundances (Figures 1A,B), PP rates at T_4_ were lower by ∼20–40% compared to the controls in the PO_4_, 2-AEPn, G6P, and PO_4_ + ATP treatments, while the rates measured in the ATP treatment bottles remained similar to the controls (Figure 3A). At the conclusion of the experiment (T_48_), PP was highest in the PO_4_ or PO_4_ + ATP amended bottles (∼50 and ∼75% higher than the controls, respectively. *p* < 0.05), while the ATP addition resulted in a weaker response (18% higher than the controls, *p* = 0.04) (Figure 3A). PP was 9 and 40% lower than the controls in the G6P and 2-AEPn amended bottles, respectively (Figure 3A). BP followed the same trend measured for heterotrophic bacterial abundance (Figure 2C). Thus, PO_4_ addition did not result in any change in BP throughout the experiment (*p* = 0.52), whereas all DOP-added treatments, including PO_4_ + ATP, triggered a significant increase at T_24_ of ∼60–100%, which diminished at T_48_ (Figure 3B). APA rates decreased in all treatments compared to the controls at T_4_–T_24_ (>50%).

## Discussion

Dissolved organic phosphorus compounds are diverse and include phospholipids, sugar-phosphates, phosphonates, nucleotides, nucleic acids, and some vitamins (Karl et al., 1998; Dyhrman et al., 2006; Clark et al., 2011). Several studies have indicated that nucleotides such as ATP are highly labile DOP molecules, as suggested by their rapid Tts (Casey et al., 2010; Orchard et al., 2010; Sebastián et al., 2012), their susceptibility to alkaline phosphatase treatment through bioassays (Berman, 1988; Bjorkman and Karl, 1994; Duhamel et al., 2018), and by specific ATP labeling (Karl and Bossard, 1985; Bossard and Karl, 1986; Karl and Björkman, 2014). Our results show that DOP is consumed by heterotrophic microbes, resulting in an increased BP, bacterial abundance and Tt, and a reduction in APA rates within 24 h (Figures 2, 3). Similarly, bioassay enrichment experiments from the NW Iberian Peninsula coast showed that heterotrophic bacteria responds faster (T_24_) than phytoplankton to additions of mixed inorganic N and P (NO_3_, NH_4_, PO_4_) and organic C and N (glucose, amino acids), than to the addition of inorganic nutrients alone (Martínez-García et al., 2010). This suggests that bacteria inhabiting the coastal water of the SEMS has the cellular machinery to utilize DOP. Moreover, DOP was fully consumed when PO_4_ was added alone or together with ATP (Figure 1 and Supplementary Table S1), a further supporting evidence that ATP is specifically an important source of P for microbial activity in the study area. The addition of PO_4_ + ATP also stimulated the highest bacterial abundance, coinciding with the highest Tt (i.e., highest relief of P-limitation). This indicates that effective DOP uptake by bacteria in the study area is intertwined with that of PO_4_, suggesting that bacteria need a source of labile organic matter to be able to utilize inorganic P.

By T_48_, BP rates returned to values close to that of the control, while PP in the PO_4_, ATP and the PO_4_ + ATP treatments increased compared to the controls, supporting the hypothesis that cyanobacteria (the main autotrophs in SEMS coastal waters), and several diatoms or dinoflagellate species, may also utilize organic molecules (discussion below). A similar inverse trend between PP and BP was also observed in the enrichment experiments of the NW Iberian Peninsula coast under mixed additions of organic and inorganic nutrients following 72 h of incubation (Martínez-García et al., 2010). This suggests that autotrophic and heterotrophic microbial communities compete for labile organic nutrients, and that in oligotrophic P-starved regions, like the SEMS, the competition is likely over organic P sources like ATP. Our results are in line with the findings of Casey et al. (2010), in a study conducted in the oligotrophic western Sargasso Sea, which demonstrated that labile DOP substances (such as ATP) constrain primary productivity in the absence of sufficient PO_4_.

P and C co-limitation of bacterioplankton in Mediterranean surface waters during seasons of vertical mixing was previously suggested by Thingstad et al. (1998), where during the non-stratified period bacteria were at times strongly C limited (Pinhassi et al., 2006). Similarly, Pinhassi and Berman (2003) performed dilution culture experiments in the eastern Mediterranean Sea finding that BP was stimulated by additions of both C and P. N and C co-limitation of bacterioplankton was also observed in the Deep Chlorophyll Maximum of the West Mediterranean (Sala et al., 2002). Finally, Rahav et al. (2016) showed that C + N + P additions to coastal SEMS water (performed in the same study site as here) stimulated BP as well as heterotrophic N_2_ fixation, while P addition alone did not. Similar to the abovementioned studies, PO_4_ additions alone to our microcosm bottles did not trigger an increase in bacterial productivity, suggesting a possible co-limitation of C and P (ATP and G6P), and likely also of N (ATP and 2-AEPn).

Alkaline phosphatases are enzymes that cleavage phosphoesters such as ATP, and thus enable the release of PO_4_ that is subsequently taken up into the cell (Jansson et al., 1988; Ammerman and Azam, 1991). Other DOP compounds may enter the bacterial cell intact via specific transport systems (i.e., no pre-cleavage is required) or by different enzymes (i.e., 5′-nucleotidases) and are therefore potentially important to bacteria (Brzoska et al., 1994). Several processes may affect the partitioning between the different species of DOP, including the release of PO_4_ from sediments, adsorption and desorption of PO_4_ from particles (i.e., dust), and microbial processes that hydrolyze organic phosphorus into PO_4_ through various enzymatic pathways (Dyhrman, 2005; Labry et al., 2016). In fact, most microorganisms can simultaneously use several metabolic pathways to utilize different DOP species, especially in oligotrophic regions where PO_4_ is scarce (i.e., the Mediterranean Sea, tropical N Atlantic, Sargasso Sea) (Karl and Björkman, 2014 and references therein). P-deficiency can therefore lead to upregulation of several genes (and thus of protein synthesis) involved in the uptake of DOP compounds like ATP (Sebastián and Ammerman, 2011; Rofner et al., 2016). For example, the enzyme 5′-nucleotidases was responsible for most of the hydrolysis of ATP in the P-amended mesocosms of east Mediterranean open water (Sebastián et al., 2012). Furthermore, a study from Californian coastal waters showed that 5′-nucleotidase activity can supply ∼50% of the PO_4_ requirements for phytoplankton in marine systems (Ammerman and Azam, 1985), suggesting 5′-nucleotidase is a key protein in DOP utilization in marine systems. Although we did not measure the activity of 5′-nucleotidase, we cannot rule out that the rapid decline in DOP concentration (Figure 1 and Supplementary Table S1) and the strong relief of P-limitation (Figures 2C,D) were attributed to both alkaline phosphatase and 5′-nucleotidase activity. Given that DOP is an important factor governing bacterial activity, we call for more dedicated studies to be conducted to follow the regulation and activity of 5′-nucleotidase and alkaline phosphatase in P-limited systems such as the oligotrophic SEMS.

Competition among microbes for limited nutrients occurs in all the oceans. This is especially true in LNLC oligotrophic regions where some of the nutrients can be found in insufficient concentrations to satisfy the metabolic requirements of the microbial communities inhabiting these waters (Amin et al., 2012). Nutrient limitation can be assessed by measuring the increase of bacterial activity following the addition of a single nutrient, or a combination of two or more nutrients (Martínez-García et al., 2010; Tsiola et al., 2016; Rahav et al., 2016, 2018). To that end, most of the studies on nutrient limitation offshore the Eastern Mediterranean Sea indicate that PO_4_ is most likely a limiting nutrient for heterotrophic bacteria (Krom et al., 1991; Zohary and Robarts, 1998; Thingstad et al., 2005). Some studies however, show that organic nutrients can also limit heterotrophic bacteria in this system (Sala et al., 2002; Martínez-García et al., 2010), highlighting the complexity of interpreting nutrient limitation for bacteria and phytoplankton in oligotrophic conditions. Our results suggest that upon DOP addition, heterotrophic bacteria outcompeted autotrophic microbial populations, resulting in an overall decrease in cyanobacteria (the dominant autotroph in the SEMS – Mella-Flores et al., 2011; Rahav et al., 2013; Hazan et al., 2018) and pico- and nano-eukaryotes abundance 4–24 h post addition (Figures 2A,B), and an increase in the heterotrophic bacterial biomass (Figure 2C). Similarly, PP decreased 4–24 h post addition, while BP significantly increased (Figures 3A,B). No significant response was recorded in these timeframes following PO_4_ addition (Figures 2, 3). Our observations correspond with a large-scale *in situ* PO_4_ fertilization experiment in the P-limited Eastern Mediterranean Sea, conducted as part of the CYCLOPS project (Thingstad et al., 2005). In that experiment, Thingstad et al. (2005) added a diluted mixture of phosphoric acid (final concentration ∼110 nmol L^–1^) and recorded a 40% decrease in chlorophyll-*a* biomass compared to control regions outside the study area, suggesting heterotrophs outcompeted autotrophs for P.

Interestingly, once the DOP limitation for heterotrophic bacteria was removed (48 h post addition), autotrophic microbial abundance (Figures 2A,B) and activity (Figure 3A) started to increase in most treatments, concurrent with the continuous decrease in DOP, but not PO_4_ (Supplementary Table S1). Many cyanobacteria, diatoms, and dinoflagellates are now recognized as mixotrophs rather than obligatory autotrophs (Béjà et al., 2000; Moore, 2013). For example, *Prochlorococcus*, one of the most common cyanobacteria on Earth (Flombaum et al., 2013), has the genetic capabilities to utilize organic substrates such as glucose (Muñoz-Marín et al., 2013). In the western tropical South Pacific Ocean, Duhamel et al. (2018) reported that both *Prochlorococcus* and *Synechococcus* can assimilate glucose, and especially leucine and ATP, due to their labile N and P content. We thus surmise that the increase in cyanobacterial abundance (*Prochlorococcus* + *Synechococcus*) and PP at the conclusion of the experiment (48 h) may be partly due to these organisms’ mixotrophic nutrition, which utilized both organic and inorganic substances. Thus, *Synechococcus* and *Prochlorococcus* may theoretically be “good” competitors of heterotrophic bacteria for labile organic molecules such as ATP and other DOP-based compounds. However, our results show that in the coastal waters of the oligotrophic SEMS, heterotrophic bacteria outcompete autotrophic microbes in the presence of elevated DOP, at least in short (4–24 h) timescales, and only when the cellular requirements of heterotrophic bacteria are met, PP increases. This is in line with the CYCLOPS findings showing that the phytoplankton compartment was bypassed by heterotrophic bacteria on phosphorus uptake (Thingstad et al., 2005). We stress that further prolonged (1 to several weeks long) studies of mixotrophic metabolism in LNLC environments, and specifically the oligotrophic SEMS, are needed, as it may have implications not only on microbial adaptations to organic and inorganic nutrient availability, but also to the marine nutrient cycling, stoichiometry and carbon drawdown.

## Data Availability

The raw data supporting the conclusions of this manuscript will be made available by the authors, without undue reservation, to any qualified researcher.

## Author Contributions

Both authors conceived and designed the experiments, performed the samplings, analyzed the data, contributed to the reagents, materials, and analysis tools, and wrote the manuscript.

## Conflict of Interest Statement

The authors declare that the research was conducted in the absence of any commercial or financial relationships that could be construed as a potential conflict of interest.

## Supplementary Material

TABLE S1 Averaged change in percent of nutrient concentrations at the conclusion of the experiment (48 h) relative to the amendment made at the beginning of the experiment (T0 h). BDL, Below Detection Limit (corresponds to a reduction of 100%). Statistically significant differences (*t*-test) are highlighted in bold.
