# Supplementary material for: DOP Stimulates Heterotrophic Bacterial Production in the Oligotrophic Southeastern Mediterranean Coastal Waters
Source: Front Microbiol. 2019 Aug 16;10:1913. doi: 10.3389/fmicb.2019.01913 (PMC6706821; doi:10.3389/fmicb.2019.01913)
Supplement: TABLE S1 — Averaged change in percent of nutrient concentrations at the conclusion of the experiment (48 h) relative to the amendment made at the beginning of the experiment (T0 h). BDL, Below Detection Limit (corresponds to a reduction of 100%). Statistically significant differences (t-test) are highlighted in bold. [file Data_Sheet_1.docx]

**Table S1**

| **Nutrient examined** | **Type of addition** | | | | | |
| --- | --- | --- | --- | --- | --- | --- |
|  | **Control** | **PO_4_** | **ATP** | **G6P** | **2-AEPn** | **PO_4_+ATP** |
| NO_2_+NO_3_ | **-65±7** | **BDL** | **BDL** | **BDL** | **BDL** | **BDL** |
| PO_4_ | -48±26 | **+17±17** | -57±28 | **+300** | -**81±15** | **+17±5** |
| Si(OH)_4_ | -5±6 | -7±9 | -7±6 | -6±9 | -1±3 | -9±10 |
| DOP | **-37±4** | **BDL** | **-37±3** | **-26±5** | +4±3 | **BDL** |
